# Removing the needle from the haystack: Enrichment of *Wolbachia* endosymbiont transcripts from host nematode RNA by Cappable-seq™

**DOI:** 10.1371/journal.pone.0173186

**Published:** 2017-03-14

**Authors:** Ashley N. Luck, Barton E. Slatko, Jeremy M. Foster

**Affiliations:** Genome Biology Division, New England Biolabs, Inc., Ipswich, MA, United States of America; International Atomic Energy Agency, AUSTRIA

## Abstract

Efficient transcriptomic sequencing of microbial mRNA derived from host-microbe associations is often compromised by the much lower relative abundance of microbial RNA in the mixed total RNA sample. One solution to this problem is to perform extensive sequencing until an acceptable level of transcriptome coverage is obtained. More cost-effective methods include use of prokaryotic and/or eukaryotic rRNA depletion strategies, sometimes in conjunction with depletion of polyadenylated eukaryotic mRNA. Here, we report use of Cappable-seq™ to specifically enrich, in a single step, *Wolbachia* endobacterial mRNA transcripts from total RNA prepared from the parasitic filarial nematode, *Brugia malayi*. The obligate *Wolbachia* endosymbiont is a proven drug target for many human filarial infections, yet the precise nature of its symbiosis with the nematode host is poorly understood. Insightful analysis of the expression levels of *Wolbachia* genes predicted to underpin the mutualistic association and of known drug target genes at different life cycle stages or in response to drug treatments is typically challenged by low transcriptomic coverage. Cappable-seq resulted in up to ~ 5-fold increase in the number of reads mapping to *Wolbachia*. On average, coverage of *Wolbachia* transcripts from *B*. *malayi* microfilariae was enriched ~40-fold by Cappable-seq. Additionally, this method has an additional benefit of selectively removing abundant prokaryotic ribosomal RNAs.The deeper microbial transcriptome sequencing afforded by Cappable-seq facilitates more detailed characterization of gene expression levels of pathogens and symbionts present in animal tissues.

## Introduction

Many filarial nematode species (including *Wuchereria bancrofti*, *Brugia malayi*, *B*. *timori* and *Onchocerca volvulus*) responsible for debilitating human diseases contain *Wolbachia*, an obligate intracellular endosymbiotic bacterium. In filarial nematodes, *Wolbachia* are found in the lateral cords of all adult worms as well as within the oocytes and developing embryos in the female reproductive tract [1–4]. Treatment with tetracycline antibiotics depletes *Wolbachia* leading to decreased fertility and eventually death of adult worms [5–7].

The precise nature of the essentiality of the *Wolbachia*-nematode symbiosis remains unclear, but, based on genomic sequences of filarial nematodes (and their respective *Wolbachia* counterparts), the obligate association is thought to derive from metabolite provisioning between the nematode and bacterium [8]. Recently, other ‘omics’ approaches (transcriptomics/proteomics) have been utilized to provide more detailed functional characterization of the mutualism between *Wolbachia* and its nematode hosts. However, studying the endosymbiont transcriptome has proven challenging as *Wolbachia* transcripts are relatively few among many nematode transcripts; similar to looking for a needle in a haystack. Next Generation sequencing has provided an unprecedented depth of sequence information that has allowed transcriptomic studies of *Wolbachia* through the sequencing of mixed total RNA (nematode + *Wolbachia*) [9–11]. While these methods offer insight on potential *Wolbachia*-host interactions, the *Wolbachia* coverage remains low. This problem is not restricted to *Wolbachia* endosymbionts of arthropods and nematodes, but to any microbial symbiont or pathogen resident in a eukaryotic host. For mixed total RNA pools, where the relative proportion of bacterial transcript is low, strategies that allow depletion of eukaryotic rRNA with or without additional steps to deplete prokaryotic rRNA and/or eukaryotic mRNA have been developed [12,13]. These approaches involve multiple manipulations to deplete different classes of eukaryotic and prokaryotic RNA that would predominate in downstream transcriptomic sequencing. Here, we employed a recently described method called Cappable-seq [14], which instead of depleting unwanted RNA classes, specifically enriches the desired *Wolbachia* mRNA transcripts from *B*. *malayi* total RNA preparations in a single step. Cappable-seq enzymatically caps 5′ triphosphorylated RNA with biotinylated GTP using the vaccinia capping enzyme [14]. Most eukaryotic mRNA contains a modified 7-methylguanylate (m^7^G) cap at the 5′-end and thus cannot be reacted with the vaccinia capping enzyme. Our use of Cappable-seq takes advantage of the fact that prokaryotic mRNA transcripts, such as those from *Wolbachia*, do not contain an m^7^G cap, but rather a 5′ triphosphate. This enables *Wolbachia* transcripts in a mixed pool of nematode/*Wolbachia* transcripts, to be selectively capped with a biotinylated GTP analog (3´ desthiobiotin-GTP), enriched on streptavidin beads and sequenced to a deeper depth than would be achievable by sequencing total nematode RNA.

## Methods

### Materials

The 3’ desthiobiotin-GTP was synthesized as previously described [14]. Adult male *B*. *malayi* and microfilariae (MF) were obtained from the peritoneal cavity of one infected jird (TRS Labs, Athens, GA, USA).

### RNA extraction

*B*. *malayi* adult males and MF were homogenized separately with ceramic beads in CK14 tubes using a Minilys homogenizer (Precellys) and total RNA was extracted by organic extraction using Trizol (Thermo-Fisher Scientific, Inc.). Samples were treated with DNase I (Thermo-Fisher Scientific, Inc.) before further Trizol extraction and final purification. RNA integrity, purity and concentration were assessed using a Bioanalyzer 2100 (Agilent Technologies).

### Vaccinia capping reaction

Total RNA from *B*. *malayi* MF (25 μg) or adult males (1.5 μg) was capped with 3´ desthiobiotin-GTP using the Vaccinia Capping System (New England Biolabs). Total RNA was incubated with 0.5 mM 3´ desthiobiotin-GTP, 0.1 mM S-adenosylmethionine (SAM) and 70 units of the Vaccinia Capping Enzyme in 1X Capping Buffer for 30 min at 37°C. RNA was then purified using the MEGAClear™ Transcription Cleanup Kit (ThermoFisher Scientific). Two additional washes (4 total washes) with the Wash Solution were performed on column to ensure complete removal of any excess 3´ desthiobiotin-GTP. To maximize recovery, RNA was eluted in 50 μL H_2_O.

### Capture of 3´ desthiobiotin capped RNA

50 μL of eluted RNA was mixed with 50 μL of 10 mM Tris-HCl, pH 7.5, 500 mM NaCl, 1 mM EDTA (wash Buffer A). This mix was added to 100 μL of hydrophilic streptavidin magnetic beads (New England Biolabs) previously washed twice with wash Buffer B (10 mM Tris-HCl, pH 7.5, 50 mM NaCl) and twice with wash Buffer A (100 μL/wash). The RNA-bead mixture was incubated for 20 min at room temperature with occasional resuspension. Using a magnetic rack, the RNA-bead mixture was washed two times with wash Buffer A, followed by two washes with wash Buffer B (100 μL/wash). All washes (containing unlabeled RNA) were collected, cleaned up using the RNeasy Kit (Qiagen) and stored at −80°C until further use (‘Uncapped RNA’ sample). Beads (containing 3´ desthiobiotin capped RNA) were resuspended in wash Buffer B containing 1 mM biotin and incubated for 20 min at room temperature with occasional mixing. After placing the tube in the magnetic rack again, the supernatant (100 μL), containing the 3´ desthiobiotin capped transcripts was collected and cleaned up using the RNA Clean and Concentrator Kit™ (Zymo), and the RNA eluted in 10 μL H_2_O and stored at −80°C until further use (‘Capped RNA’ sample).

### Library preparation and sequencing

Prior to library construction, the RNA integrity, purity and concentration of all samples were assessed on an RNA pico or nano chip using a Bioanalyzer (Agilent). Libraries were prepared from capped RNA, uncapped RNA (from bead wash steps above) and total RNA (12.9 ng/1.5 ng, 100 ng/100 ng and 100 ng/70 ng from *B*. *malayi* MF/adult males, respectively), using the NEBNext^®^ Ultra™ Directional RNA Library Prep Kit for Illumina^®^ (New England Biolabs) according to the manufacturer instructions. Library quality was assessed using a DNA high sensitivity chip on a Bioanalyzer 2100 prior to sequencing. The three libraries (total, capped and uncapped) for each sample (MF and adult male) were pooled and were sequenced on an Illumina NextSeq (75 bp single-end reads).

### Bioinformatic analysis

All data was analyzed using a local instance of Galaxy [15–17]. Sequence reads from each sample were first trimmed and assessed for quality using Sickle (Galaxy v 1.0.0, default settings) [18] and FastQC (Galaxy v 1.0.0) [19]. RNA-Seq reads from each sample were mapped to the genome of the *Wolbachia* endosymbiont of *B*. *malayi*, *w*Bm (version 2.2) or to the *B*. *malayi* genome (Wormbase version WS236) using the default settings of Bowtie2 (Galaxy v 0.2) [20]. Reads aligned using Bowtie2 were assembled into transcripts using the default settings of Cufflinks (Galaxy v 0.0.7) [21].

## Results and discussion

A total of over 137 million reads were sequenced from *B*. *malayi* adult male and microfilarial (MF) transcriptomic libraries. Over 6 million of these reads mapped to the *Wolbachia* endosymbiont (Table 1). While the percentage of reads mapping to *B*. *malayi* in the six RNA samples was relatively unchanged (Table 2) likely due to non-specific binding to the streptavidin beads, the percentage of reads mapping to *Wolbachia* was enriched in the samples treated with Cappable-seq (Table 1). Prior sequencing of total RNA from different lifecycle stages of a similar filarial nematode, *Dirofilaria immitis*, yielded ~0.7% average reads mappable to *Wolbachia* (0.02–2.11% depending on life-cycle stage) [10]. Similarly, in the present study between 1.3% (adult male) and 1.6% (MF) of reads from *B*. *malayi* total RNA mapped to the *w*Bm genome (Table 1). Comparatively, 4.9% (adult male) and 7.6% (MF) of the RNA captured by the Cappable-seq technique mapped to the *w*Bm genome. This represents a 3.8-fold (adult male) or 4.8-fold (MF) enrichment in *Wolbachia* transcripts using Cappable-seq (Table 1). Additionally, the increased percentage of reads mapping to *Wolbachia* does not appear to be due to the higher number of sequenced reads in the capped library because downsampling of both the capped and total RNA libraries to 10 million reads had no effect on the percentage of reads mapping to *Wolbachia* (S1 Table).

**Table 1:** Total number of reads sequenced and mapped to *Wolbachia* from *B*. *malayi* (*w*Bm).

RNA	Reads (Total)	Reads Mapped to *w*Bm	*w*Bm % Total Reads	Fold Increase in % Reads Mapping to *w*Bm (vs. total RNA)
Microfilarial Total	18,381,038	291,425	1.6%	-
Microfilarial Capped	46,196,181	3,489,630	7.6%	4.8
Microfilarial Uncapped	7,742,516	132,789	1.7%	1.1
Adult Male Total	16,938,674	216,409	1.3%	-
Adult Male Capped	39,313,992	1,938,538	4.9%	3.8
Adult Male Uncapped	9,357,191	99,262	1.1%	0.8

**Table 2:** Total number of reads sequenced and mapped to *B*. *malayi*.

RNA	Reads (Total)	Reads Mapped to *B*. *malayi*	*B*. *malayi* % Total Reads	Fold Increase in % Reads Mapping to *B*. *malayi* (vs. total RNA)
Microfilarial Total	18,381,038	13,643,089	74.2%	-
Microfilarial Capped	46,196,181	36,191,120	78.3%	1.1
Microfilarial Uncapped	7,742,516	5,724,095	73.9%	1.0
Adult Male Total	16,938,674	12,918,089	76.3%	-
Adult Male Capped	39,313,992	29,354,897	74.7%	1.0
Adult Male Uncapped	9,357,191	6,940,809	74.2%	1.0

As expected, the uncapped transcripts are not enriched for *Wolbachia* transcripts as compared to the total RNA control (1.7% vs 1.6% and 1.1% vs 1.3%, for MF and adult male samples, respectively, Table 1).

**Fig 1:**
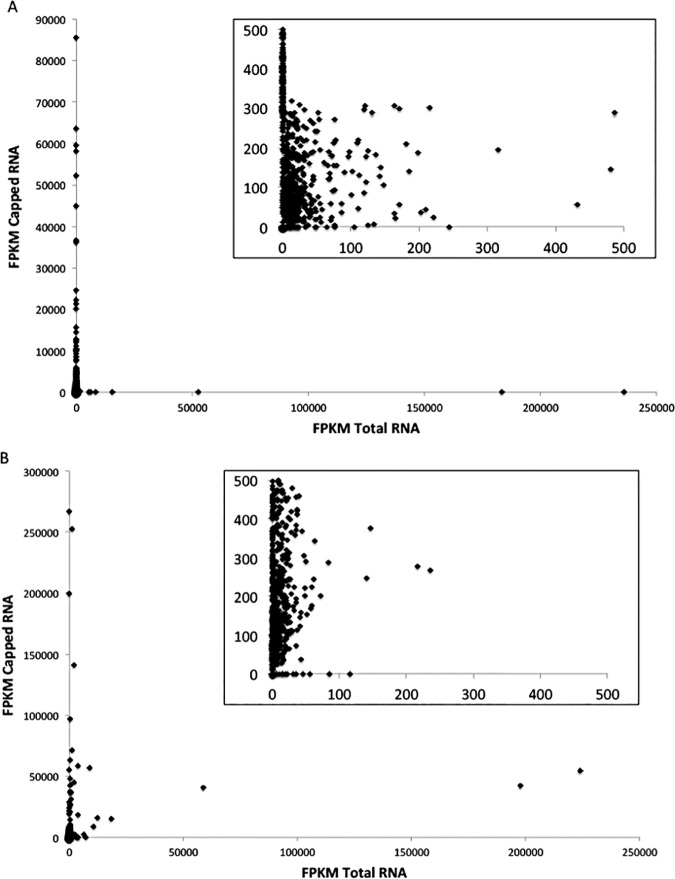
**A.** Transcript coverage (FPKM) of *Wolbachia* genes reveals over 88% of *Wolbachia* transcripts from *B*. *malayi* adult male RNA were enriched using the Cappable-seq technique. A closer view of transcript abundance (inset) reveals most *Wolbachia* transcripts in total RNA are present in very low abundance, whereas the *Wolbachia* transcripts are more abundant in the capped RNA sample. Points along the y-axis are indicative of *Wolbachia* transcripts that were undetectable in total RNA that were detected in the capped RNA sample. **B.** Transcript coverage (FPKM) of *Wolbachia* genes reveals over 95% of *Wolbachia* transcripts from *B*. *malayi* MF RNA were enriched using the Cappable-seq technique. A closer view of transcript abundance (inset) reveals most *Wolbachia* transcripts in total RNA are present in very low abundance, whereas the *Wolbachia* transcripts are more abundant in the capped RNA sample. Points along the y-axis are indicative of *Wolbachia* transcripts that were undetectable in total RNA that were detected in the capped RNA sample.

Reads mapping to *w*Bm were assembled into transcripts and quantified into FPKM values (Fragments Per Kilobase of transcript per Million mapped reads) to assess whether transcriptomic coverage was enriched with Cappable-seq. Current annotation suggests the *w*Bm genome contains 940 predicted transcripts, including ribosomal RNAs (rRNA) and tRNAs. Total RNA sequencing covered between ~58–69% of the *w*Bm transcriptome with 544/940 (adult male) and 649/940 (MF) transcripts having FPKM>0 (Fig 1A and 1B, Table 3, S2 and S3 Tables). Captured RNA, following Cappable-seq, covered ~91–97% of the transcriptome with 852/940 (adult male) or 909/940 (MF) of transcripts detected (Fig 1A and 1B, Table 3, S2 and S3 Tables). While this may seem surprising that nearly all *Wolbachia* transcripts would be expressed, this is not unexpected with an intracellular bacterium such as *Wolbachia* that has a highly reduced, ‘minimal’ genome of only ~800 coding genes, versus thousands of coding genes in most bacteria. Moreover, high coverage across the *Wolbachia* transcriptome has been previously reported without any prokaryotic transcript enrichment for *Wolbachia* from *Onchocerca ochengi* [9] and for *Wobachia* from *Dirofilaria immitis* [10,11]. Importantly, however, in this study, high *Wolbachia* transcriptome coverage was achieved with less overall sequencing. Additionally, although not directly tested in this study, the increased coverage of the *Wolbachia* transciptome using Cappable-seq would more likely allow more comprehensive differential gene expression analysis as compared to sequencing total RNA.

**Table 3:** *Wolbachia* Transcript Coverage Increases by using Cappable-seq.

RNA	# Transcripts Covered (FPKM>0)	% Transcripts Covered (n = 940)	Average FPKM/Transcript	Fold Increase in Average FPKM/Transcript
Microfilarial Total	649	69.0%	632	-
Microfilarial Capped	909	96.7%	2934	4.6
Adult Male Total	544	57.9%	562	-
Adult Male Capped	852	90.6%	1161	2.1

**Fig 2:**
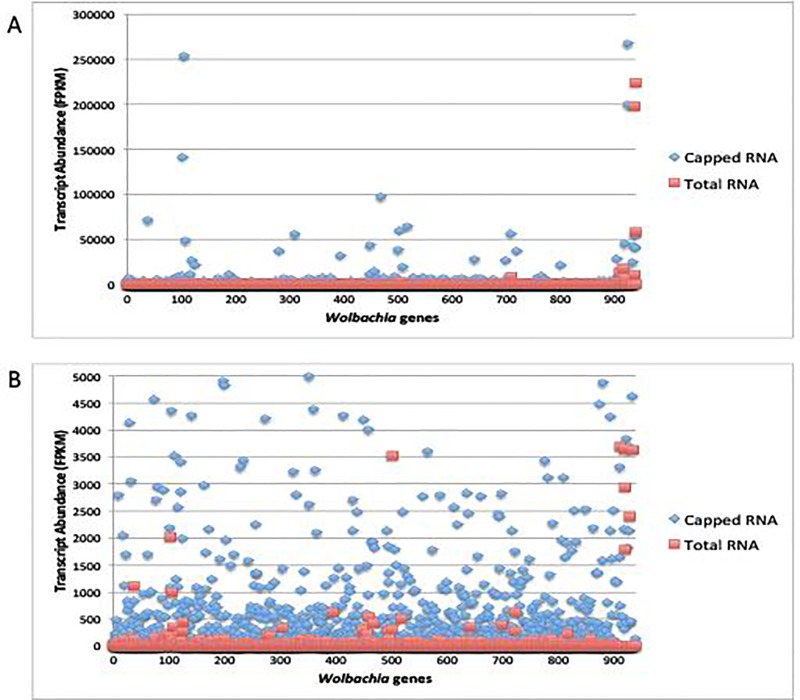
(A) Transcript abundance of all 940 annotated *Wolbachia* genes (listed by geneID number) reveals over 95% of *Wolbachia* transcripts from *B*. *malayi* MF RNA were enriched using the Cappable-seq technique. Each transcript is indicated by in red (the FPKM value in the total RNA) and blue (the FPKM value in capped-RNA sample)(B) A closer view (note difference in y-axis scales between panel A and B) of transcript abundance reveals an enrichment in *Wolbachia* transcripts in the capped RNA sample.

**Fig 3:**
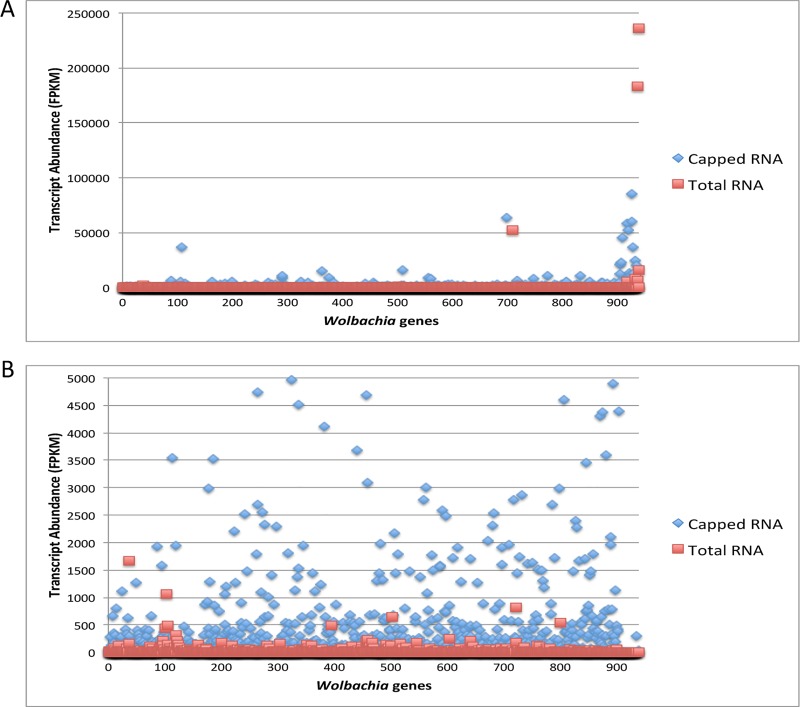
(A) Transcript abundance of all 940 annotated *Wolbachia* genes (listed by geneID number) reveals over 95% of *Wolbachia* transcripts from *B*. *malayi* adult male RNA were enriched using the Cappable-seq technique. Each transcript is indicated by in red (the FPKM value in the total RNA) and blue (the FPKM value in capped-RNA sample)(B) A closer view (note difference in y-axis scales between panel A and B) of transcript abundance reveals an enrichment in *Wolbachia* transcripts in the capped RNA sample.

Importantly, an additional 276 *Wolbachia* transcripts (29.4% of the total transcriptome) in the *B*. *malayi* MF sample and 334 *Wolbachia* transcripts (35.5% of the total transcriptome) in the *B*. *malayi* adult sample were detected as expressed using Cappable-seq that were not detected by sequencing total RNA (Fig 1A and 1B, Table S2). Not only were more transcripts detected, but also average transcript coverage (average FPKM value/transcript) was increased 2-fold (adult male) or 4-fold (MF) with Cappable-seq (Figs 2 and 3, Table 3).

Comparison of the FPKM values between the capped and total RNA samples (FPKM Capped/FPKM Total) reveals an average enrichment of ~10 to 40-fold in adult male worms (n = 518 transcripts with FPKM values in both capped and total samples) and MF (n = 633 transcripts with FPKM values in both capped and total samples), respectively (S2 and S3 Tables). This enrichment value is likely underrepresented in the adult male sample due to the fact that fewer transcripts were detected in the adult male total RNA than the MF total RNA (because this enrichment value can only be derived if the transcript was detected in both the capped and total RNA samples). Additionally, it is unlikely this increase is due to the higher number of sequenced reads in the capped library because the FPKM value is normalized based on the number of reads sequenced (Fragments Per Kilobase of transcript per Million mapped reads).

Interestingly, some tRNAs transcripts were enriched by Cappable-seq (S2 and S3 Tables). It is unclear exactly why some *Wolbachia* tRNAs may be enriched by Cappable-seq while others are depleted. Furthermore, a number of pseudogenes were also enriched by Cappable-seq (S2 and S3 Tables). However, widespread expression of *Wolbachia* pseudogenes was previously noted in *Wolbachia* from *Onchocerca ochengi* [9], and importantly, transcript expression does not verify a functional gene product.

Some *Wolbachia* transcripts were actually depleted by using Cappable-seq, but this is not unexpected. For example, ribosomal RNA is rapidly processed in the cell and therefore would not be a substrate for the vaccinia capping enzyme. Application of Cappable-seq to *E*.*coli* RNA reduced the number of reads that mapped to rRNA from 85% to just 3% [14]. In the adult male sample, we see complete depletion (0 FPKM) for all three *Wolbachia* ribosomal RNA transcripts, while in the MF sample, we observe a 79% depletion of the *Wolbachia* 23S rRNA gene, a 32% depletion of the *Wolbachia* 5S rRNA gene and a 76% depletion of the *Wolbachia* 16S rRNA gene (S3 Table). Additionally, there is widespread lateral gene transfer (LGT) from *Wolbachia* to its eukaryotic hosts, including *B*. *malayi*, and LGT transcription from these host genomes is known [22,23]. If these transcripts were the product of eukaryotic pol II, they would bear a 5´ m^7^G cap and also be depleted by Cappable-seq.

A number of metabolic relationships potentially responsible for the mutualism between *B*. *malayi* and *Wolbachia* have been proposed based on the genomes of the two organisms [8,24,25]. Filarial nematodes, such as *B*. *malayi*, lack genes required for *de novo* synthesis of purines, pyrimidines and other cofactors (heme and riboflavin), while these pathways are complete and likely functional within their *Wolbachia* endosymbiont [8]. Moreover, given the essentiality of *Wolbachia* for nematode survival, targeting of these essential *Wolbachia* pathways may have detrimental effects on their parasitic nematode hosts. Unfortunately, transcriptomic analysis of these essential *Wolbachia*-specific, potentially therapeutic pathways, is often hindered by sequencing total RNA due to low transcript coverage of one or more genes in the pathway. Although gene expression alone does not confirm translation or gene functionality, transcript coverage of nearly every gene in the purine, pyrimidine, heme and riboflavin biosynthesis pathways was enriched using Cappable-seq. Importantly, transcripts for over one third (17/45) of the *Wolbachia* genes in these pathways were undetectable in *B*. *malayi* adult male total RNA, but were detected using Cappable-seq. Godel *et al*. [26] suggested a number of *D*. *immitis Wolbachia* genes that may be suitable drug targets. Specific targets that have been suggested include proteins involved in nucleic acid synthesis (DnaB), enzymes involved in fatty acid synthesis (FabZ) [26], and the previously identified *Wolbachia* target, FtsZ, a cell division protein [27]. Of these targets, DnaB and FtsZ were enriched (~21-23-fold and ~12-27-fold, respectively) and FabZ went from undetectable in total RNA to detected in capped RNA. Transcripts for other proposed *Wolbachia*-specific drug targets including pyruvate phosphate dikinase (PPDK) [28] and cofactor-independent phosphoglycerate mutase (iPGM) [29] were also enriched (3 to 28-fold). Thus, Cappable-seq can be used to gather more meaningful information on the expression of these pathways and potential *Wolbachia* targets in the host-endosymbiont interaction.

### Conclusions

We have used a modified Cappable-seq protocol to enrich *Wolbachia* transcripts from microfilarial and adult male *B*. *malayi* total RNA. We selected these different life-cycle stages since adult males typically represent a small sample size with a low level of *Wolbachia* transcription compared to the larger MF samples that have also much higher endosymbiont transcriptional activity [10]. Using these samples, we obtained a ~4 to 5-fold enrichment of *Wolbachia* transcripts, corresponding to an average 10 to 40-fold enrichment in *Wolbachia* transcripts that were detected both before and after Cappable-seq. We also observed significant depletion of *Wolbachia* rRNA transcripts following Cappable-seq. The levels of enrichment of *Wolbachia* transcripts from MF and adult males were thus broadly similar despite a nearly 20-fold difference in the amount of total RNA used for Cappable-seq. The levels of *Wolbachia* transcript enrichment obtained by Cappable-seq surpass those obtained by alternative strategies that use combined rRNA and polyA depletion approaches [13]. Our use of the technology to enrich *Wolbachia* endosymbiont transcripts from a large background of host nematode RNA allows deeper sequencing of the *Wolbachia* transcriptome with reduced sequencing effort and cost. With further optimization of parameters, we anticipate that even greater levels of enrichment might be realized. This method should prove applicable to other mixed RNA transcript samples that contain relatively few uncapped prokaryotic endosymbiont or pathogen RNAs in a pool of multitudinous capped eukaryotic RNAs and allow for more robust differential expression analysis of prokaryotic transcripts.

## Supporting information

S1 Table Downsampling to 10 million sequencing reads does not effect the percentage of read mapping to *Wolbachia* from *B*. *malayi* (*w*Bm). (DOCX)

S2 Table *Wolbachia* Transcript Quantitation from Capped and Total RNA from Adult Male *B*. *malayi*. (XLSX)

S3 Table *Wolbachia* Transcript Quantitation from Capped and Total RNA from *B*. *malayi* Microfilariae. (XLSX)
