# Supplementary material for: Removing the needle from the haystack: Enrichment of Wolbachia endosymbiont transcripts from host nematode RNA by Cappable-seq™
Source: PLoS One. 2017 Mar 14;12(3):e0173186. doi: 10.1371/journal.pone.0173186 (PMC5349465; doi:10.1371/journal.pone.0173186)
Supplement: S1 Table — (DOCX) [file pone.0173186.s001.docx]

Table S1: Downsampling to 10 million sequencing reads does not effect the percentage of read mapping to *Wolbachia* from *B. malayi* (*w*Bm).

| **RNA** | **Reads (Total)** | **Reads Mapped to *w*Bm** | ***w*Bm % Total Reads** | **Fold Increase in % Reads Mapping to *w*Bm (vs. total RNA)** |
| --- | --- | --- | --- | --- |
| Microfilarial Total | 18,381,038 | 291,425 | 1.6% | - |
| Microfilarial Capped | 46,196,181 | 3,489,630 | 7.6% | 4.8 |
| Downsampled to  10 million reads |  |  |  |  |
| Microfilarial Total | 10,000,000 | 160,215 | 1.6% | - |
| Microfilarial Capped | 10,000,000 | 786,079 | 7.9% | 4.9 |
|  |  |  |  |  |
| Adult Male Total | 16,938,674 | 216,409 | 1.3% | - |
| Adult Male Capped | 39,313,992 | 1,938,538 | 4.9% | 3.8 |
| Downsampled to  10 million reads |  |  |  |  |
| Adult Male Total | 10,000,000 | 129,118 | 1.3% | - |
| Adult Male Capped | 10,000,000 | 567,788 | 5.7% | 4.4 |
